# Executive functioning and spoken language skills in young children with hearing aids and cochlear implants: Longitudinal findings

**DOI:** 10.3389/fpsyg.2022.987256

**Published:** 2022-09-23

**Authors:** Izabela A. Jamsek, William G. Kronenberger, David B. Pisoni, Rachael Frush Holt

**Affiliations:** ^1^Department of Speech and Hearing Sciences, Ohio State University, Columbus, OH, United States; ^2^Department of Psychiatry, Indiana University School of Medicine, Indianapolis, IN, United States; ^3^DeVault Otologic Research Laboratory, Department of Otolaryngology-Head and Neck Surgery, Indiana University School of Medicine, Indianapolis, IN, United States; ^4^Department of Psychological and Brain Sciences, Indiana University, Bloomington, IN, United States

**Keywords:** hearing loss, children, executive functioning, language, hearing aids, cochlear implants

## Abstract

Deaf or hard-of-hearing (DHH) children who use auditory-oral communication display considerable variability in spoken language and executive functioning outcomes. Furthermore, language and executive functioning skills are strongly associated with each other in DHH children, which may be relevant for explaining this variability in outcomes. However, longitudinal investigations of language and executive functioning during the important preschool period of development in DHH children are rare. This study examined the predictive, reciprocal associations between executive functioning and spoken language over a 1-year period in samples of 53 DHH and 59 typically hearing (TH) children between ages 3–8 years at baseline. Participants were assessed on measures of receptive spoken language (vocabulary, sentence comprehension, and following spoken directions) and caregiver-completed executive functioning child behavior checklists during two in-person home visits separated by 1 year. In the sample of DHH children, better executive functioning at baseline (Time 1) was associated with better performance on the higher-order language measures (sentence comprehension and following spoken directions) 1 year later (Time 2). In contrast, none of the Time 1 language measures were associated with better executive functioning in Time 2 in the DHH sample. TH children showed no significant language-executive functioning correlations over the 1-year study period. In regression analyses controlling for Time 1 language scores, Time 1 executive functioning predicted Time 2 language outcomes in the combined DHH and TH samples, and for vocabulary, that association was stronger in the DHH than in the TH sample. In contrast, after controlling for Time 1 executive functioning, none of the regression analyses predicting Time 2 executive functioning from Time 1 language were statistically significant. These results are the first findings to demonstrate that everyday parent-rated executive functioning behaviors predict basic (vocabulary) and higher-order (comprehension, following directions) spoken language development 1 year later in young (3–8 year old) DHH children, even after accounting for initial baseline language skills.

## Introduction

Children who are deaf or hard-of-hearing (DHH) and use hearing aids (HAs) or cochlear implants (CIs) for auditory-oral communication display considerable variability in language and neurocognitive outcomes (Niparko et al., 2010; Kronenberger et al., 2014; McCreery and Walker, 2022). Neurocognitive contributors to language outcomes are of considerable interest to researchers and clinicians because they offer a potential intervention target that may help to explain and improve language outcomes in the DHH population (Eisenberg et al., 2007; Pisoni et al., 2018). One domain of neurocognitive functioning that may support language development in DHH populations is executive functioning (EF; Kronenberger and Pisoni, 2020). EF encompasses a broad set of neurocognitive abilities required to actively control thought, behavior, and emotion in order to remain focused and goal-directed (Barkley, 2012). EF is composed of neurocognitive skills in distinct, yet interrelated, domains including working memory, inhibition, and shifting (Miyake et al., 2000). Working memory involves the capacity to retain and manipulate novel information (Barkley, 2012). Inhibition refers to the ability to resist and overcome initial impulsive, prepotent responses to achieve goal-directed behavior (Barkley, 2012). Shifting encompasses the ability to adjust to novel or competing stimuli in problem-solving (Barkley, 2012). Working memory has been the most-frequently investigated EF domain involved in language processing and learning in typically hearing (TH) children (e.g., Baddeley, 2003). Working memory is thought to enable and maximize in-the-moment language processing that supports higher-order language development (Carpenter and Just, 2013) as well as play a reciprocal role in vocabulary learning and retention (Gathercole and Baddeley, 1993). Inhibitory control has been related to higher-order language grammatical ability in TH children through its proposed role in active evaluation of language and grammatical rules during language processing (Ibbotson and Kearvell-White, 2015). Conversely, inhibition has been implicated as an outcome of robust vocabulary development in TH toddlerhood providing a scaffold to strengthen inhibitory skills (Chow et al., 2019). EF domains are interrelated with each other and with language throughout development, but especially in children. Indeed, previous research has supported fewer functional, measurable differences in EF domains earlier in development (Messer et al., 2018). For example, shifting has not been supported as a separate, measurable construct for performance-based EF tasks until later school-age (Messer et al., 2018). Some research has also suggested that working memory and inhibition are most strongly associated in younger children and then become more distinct and less tightly associated as children age (Lerner and Lonigan, 2014). Examination of related domains of child EF skills in fewer or single constructs representing children’s overall functioning is therefore, a common practice in the field of developmental research (e.g., Gooch et al., 2016).

Longitudinal evidence in preschool and early elementary years in TH children has found that EF and language are strongly related concurrently and largely resilient to influence over time (Gooch et al., 2016). Increasing evidence supports the findings that EF and language in TH children may exhibit smaller transactional and bidirectional effects at least to age 5 (Fuhs et al., 2014; Weiland et al., 2014; Slot and von Suchodoletz, 2018). One avenue by which EF is hypothesized to play a role in scaffolding language development is by providing attentional and behavioral support to maximize language learning opportunities (Bishop et al., 2014).

In DHH children, there is emerging evidence that EF may play an outsized role during language processing and learning relative to TH children, due to challenges in listening effort, quality of language exposure, and underspecified phonological-lexical representations of words in short- and long-term memory associated with hearing loss (Rönnberg et al., 2013; Pichora-Fuller et al., 2016; Kronenberger et al., 2018). Even with best-fit hearing technology and appropriate and timely early intervention, DHH children, on average, exhibit language delays, incomplete or underspecified phonological and lexical representations, and weaker lexical networks for spoken words (Pisoni et al., 2011). Any interruptions or difficulties with the underlying auditory and linguistic skills needed to process language increases the cognitive effort involved (Beckage et al., 2011; Kenett et al., 2013), placing greater demands on EF in language processing tasks (Kronenberger et al., 2018). In fact, in a study with a dual-task paradigm designed to vary cognitive load and EF demands, DHH children displayed greater language decrements when they had fewer EF resources available to support language processing, compared to TH peers (Kronenberger et al., 2018). Even for tasks with smaller EF loads, the language processing of DHH children was found to be slower and more effortful than that of TH children, as a result of DHH children’s use of EF skills as a compensatory strategy to successfully encode and process language (Kronenberger et al., 2018). In DHH children, EF skills may not simply maximize available learning opportunities, as hypothesized in TH children, but operate as a mandatory skill to overcome consistent language processing difficulties to enable language learning. Hypotheses positing the opposite causal direction, that language abilities facilitate later EF skills, suggest that language may serve as a method to facilitate control of behavior when the environment or task demands make successful EF performance difficult (Zelazo and Frye, 1998). Thus, although the co-development of language and EF across childhood is very likely reciprocal, bidirectional, and important for childhood development (Bohlmann et al., 2015), EF and language may be more tightly linked in DHH children than in TH children (Kronenberger et al., 2018).

Most of the current evidence supporting an association between EF and language skills in DHH children has been obtained using cross-sectional methods (e.g., Botting et al., 2017; McCreery and Walker, 2022). Predictive, causal, and mechanistic claims cannot be fully supported by cross-sectional data, warranting more longitudinal investigations. However, longitudinal investigations of EF and language during the critical preschool period of rapid, early language development are rare in DHH children (Kronenberger et al., 2020), with most cross-sectional and longitudinal studies focusing on school ages or older (e.g., Jones et al., 2020), after most of the significant, early, transactional effects of language and EF may have already occurred. For example, Jones et al. (2020) evaluated DHH and TH children aged 6–11 at first test and 2 years later using individually administered behavioral measures of expressive vocabulary and lab/performance-based EF. They found that expressive vocabulary significantly predicted later EF for a majority of EF tasks, but not the reverse. An earlier study by Harris et al. (2013) evaluated DHH children in the same age range at first test (6–11 years) and found the opposite result: performance-based EF (specifically, working memory) predicted both receptive vocabulary and higher-order language ability over a range of 1.5–4.5 years later. A recent study with a younger group of DHH and TH children (3–6 years at first study visit) over a period of 1–5 years showed that EF predicted vocabulary and global language 1 year later, but language only predicted later verbal short-term memory, as opposed to inhibition, shifting, or parent-rated executive functioning behaviors (Kronenberger et al., 2020).

In addition to behavioral measures, Kronenberger et al. (2020) also included a parent-rated, questionnaire-based measure of EF in analyses, the Behavior Rating Inventory of Executive Functioning (BRIEF; Gioia et al., 2000). Measuring EF using parent-rated behaviors observed in the child’s daily life may add ecological validity to understanding associations between language and EF and their expression in real life for the development of interventions (Kronenberger et al., 2014; Castellanos et al., 2018), given that individually administered neurocognitive measures of EF correlate only modestly with actual EF behaviors in the day-to-day environment (Barkley, 2012). When BRIEF scales of Working Memory, Inhibit, and Shift were added into the predictive models, BRIEF Shift significantly predicted later vocabulary scores only in the DHH sample, not in TH children (Kronenberger et al., 2020). Recent cross-sectional investigations further explored this pattern of findings that language and EF of DHH children may exhibit stronger associations than TH children of the same age (Blank et al., 2020; Jamsek et al., 2021).

Thus, while existing research suggests associations between EF and language that may be stronger in DHH than TH samples, methodological limitations constrain the current body of knowledge. Cross-sectional/concurrent studies comprise the vast majority of language-EF research with DHH children but cannot address longitudinal, predictive, or causal influences. Longitudinal research has significant advantages, but very few longitudinal studies have been undertaken in this area, all of which have additional limitations. For example, only one longitudinal study (Jones et al., 2020) controlled for baseline language or EF when predicting later language or EF scores. Controlling for baseline values of an outcome variable (as in cross-lagged analyses) has the advantage of removing the effects of the baseline (concurrent) correlation between two variables (in this case, language and EF) when testing the longitudinal association between the variables. However, that strategy may mask earlier causal relationships between the variables in question, which created the baseline (concurrent) correlation in the first place. As a result, earlier reciprocal or unidirectional influences between language and EF may be responsible for a strong concurrent association found later in development, and removing that later concurrent association obscures the earlier EF-language effect. The best way to address this latter issue may be to investigate language-EF influences at very young ages when children are in a period of rapid development and change. Studies at later ages, even early to middle school ages, may miss the critical early influences transacting between language and EF that occur during preschool and very early school ages, particularly when controlling for baseline language and EF. Finally, very little research has investigated EF in the child’s daily behavior, based on parent-report. While parent-reports of child behavior have well-known limitations (Toplak et al., 2013; Friedman and Gustavson, 2022), compared to lab/performance-based measures they are also a much more ecologically valid assessment of the child’s EF in daily life (Barkley, 2012), which would be expected to more closely correspond to language exposure and processing.

The objective of this study was to examine the predictive, reciprocal associations of EF on language and language on EF over a 1-year period in samples of preschool- and early school age DHH and TH children (3–8 years of age; henceforth referred to as “preschool-age” for simplicity and recognition that many DHH children enter early school grades at slightly later ages). This study extends previous research by incorporating parent-report questionnaire measures of EF, controlling for baseline language/EF measures in analyses, and assessing multiple domains of language in a sample of young children. Study hypotheses were as follows: (1) Preschool-aged children will demonstrate reciprocal, longitudinal associations between EF and language after accounting for baseline EF/language skills; and 2) those associations will be stronger for DHH than for TH children.

## Materials and methods

### Participants and procedure

One hundred and twelve children between the ages of 3–8 years at their first study visit participated in a larger longitudinal study of family environment and developmental outcomes in children who are DHH and primarily use auditory-oral communication (Families and Hearing Study; Holt et al., 2020). All participants were screened for non-verbal reasoning ability on the Differential Ability Scales–Second Edition Picture Similarities subtest (Elliott et al., 2007), and were included in the study if they scored higher than 2 standard deviations below the mean (*T*-score > 30). In addition, each child’s primary caregiver reported typical hearing, English as the primary home language, and no history of developmental disabilities/delays in their child (other than known sequelae of hearing loss in the DHH sample). Inclusion criteria for all TH children included passing a bilateral behavioral hearing screening at 25 dB HL at octave frequencies between and including 250–4,000 Hz (re: American National Standards Institute, 2010) at their first visit. The screening was administered by clinical researchers in the families’ homes using an Earscan 3 handheld screening audiometer with insert earphones (Micro Audiometrics Corporation, 2018). DHH children were included if they had a bilateral, sensorineural hearing loss that was identified before 2 years of age and received intervention with amplification (HAs or CIs) before 2 years of age. The children with CIs were implanted before 3.5 years of age, the majority before 3 years.

The DHH sample was primarily recruited from hospital databases of DHH children with HAs and/or CIs in Ohio and Indiana. Both TH and DHH children were also recruited via online and hard copy recruitment posters in the surrounding communities, including medical settings and organizations serving both TH and DHH children. Participants completed two in-person research home visits [Time 1 (T1) and Time 2 (T2)] separated by 10–14 months. During home visits, which typically lasted 2.5 h, one clinical researcher administered child assessments (including spoken language measures), while the other worked with the child’s primary caregiver, who also completed study questionnaires just before each visit (including EF, background/demographic, and hearing history questionnaires). Fifty-five of the TH child caregivers were mothers and four were fathers. Forty-three of the DHH child caregivers were mothers, three were fathers, five were adoptive mothers, and two were grandmothers. The sample (demographics displayed in Table 1) was composed of 59 TH children and 53 DHH children (24 HA users and 29 CI users) with no significant differences in gender composition between hearing groups. The TH sample was significantly younger and had significantly higher levels of parental education and annual family income than the DHH sample (Table 1). Children who used bilateral HAs had a significantly longer amount of time since first device fit and better hearing as measured by lower unaided better ear 4-frequency pure-tone average (PTA) than children who used at least one CI (Table 1).

**TABLE 1:** T1 participant demographics and audiological characteristics.

	TH	DHH (HA and CI)	HA	CI
Characteristics	M (SD)	M (SD)	M (SD)	M (SD)
Demographics				
N	59	53	24	29
N females/males	27/32	27/26	12/12	15/14
Chronological age, child (years)	5.78 (1.61)	6.55* (1.55)	6.55 (1.71)	6.55 (1.43)
Parental education^a^	8.12 (1.26)	7.66* (1.22)	7.75 (1.15)	7.59 (1.30)
Annual family income^b^	8.81 (1.58)	7.77* (2.64)	8.33 (2.12)	7.31 (2.95)
Audiological characteristics				
Hearing age (years)^c^	n/a	4.88 (1.94)	5.61 (1.75)	4.27* (1.90)
Unaided 4-frequency PTA^d^ (dB HL)	n/a	72.3 (28.59)	50.21 (15.06)	92.64*** (22.24)
Aided 4-frequency PTA^e^ (dB HL)	n/a	23.51 (6.41)	21.14 (9.82)	24.52 (4.12)

Independent samples t-tests were used to compare between hearing groups; N, number of participants; TH, typical hearing; DHH, deaf or hard-of-hearing; HA, hearing aid; CI, cochlear implant; PTA, pure-tone average re: American National Standards Institute (2004); n/a = not applicable. ^a^Parental education was coded based on highest level of formal education: 1 = elementary school through 10 = doctorate degree. ^b^Parents indicated their annual income on a 1 (under $5,000) to 10 ($95,000 and over) scale. ^c^Calculated by subtracting age at which child was first fit with HAs or CIs from their chronological age. ^d^Calculated at 0.5, 1, 2, and 4 kHz in the better ear based on data from 50 children (24 HA and 26 CI users, respectively) due to lack of access to the medical information for a subset of children. ^e^Calculated at 0.5, 1, 2, and 4 kHz in the better ear based on data from 37 children (11 HA and 26 CI users, respectively). *p < 0.05, ***p < 0.001.

### Measures

#### Receptive spoken language

Children were individually administered three measures designed to assess different domains of receptive language: single-word vocabulary [Peabody Picture Vocabulary Test-4 (PPVT); Dunn and Dunn, 2007], sentence comprehension [Comprehensive Assessment of Spoken Language-2 (CASL) Sentence Comprehension subtest; Carrow-Woolfolk, 2017], and following spoken directions [Clinical Evaluation of Language Fundamentals (CELF) Following Directions subtest; Wiig et al., 2004; Semel et al., 2013]. The CELF-Preschool-2 Concepts and Following Directions subtest (Semel et al., 2013) was used for children ages 3–5 years and the CELF-5 Following Directions subtest (Wiig et al., 2004) was used for children ages 6 years and older. Receptive language measures were chosen to reduce task demands and avoid scoring ambiguity from potentially distorted speech in children who are DHH, because these receptive measures do not require a verbal response from the participant. The PPVT is a widely used, normed measure of single-word receptive vocabulary that requires participants to identify a picture from among a set of four choices that corresponds to a word spoken aloud by the examiner. The CASL Sentence Comprehension subtest requires participants to indicate the picture that corresponds to a sentence spoken by the experimenter. If participants reach the end of that section, they are also asked to evaluate a pair of sentences spoken by the experimenter for their semantic equivalence. The CELF Following Directions subtest requires participants to sequentially point to items indicated by the experimenter in directive sentences of increasing length and complexity. Scoring for all three language measures includes standard scores (scaled scores in the case of the CELF) based on their respective normative samples, in which higher scores correspond to better receptive spoken language ability. These measures were chosen to assess distinct areas of language learning under active development in early school-age children. Single-word vocabulary is a basic building block of language development, while sentence comprehension and following directions are considered higher-order language processes. The CASL is a broader measure of the stage of language development, while the CELF involves attentional components that could implicate EF skills to a greater degree.

#### Executive functioning behavior checklists

Caregivers completed the Behavior Rating Inventory of Executive Functioning (BRIEF; BRIEF-Preschool for 3–5 years and BRIEF-2 for 6 + years; Gioia et al., 1996, 2015) and the Learning, Executive, and Attention Functioning scale (LEAF; Castellanos et al., 2018). BRIEF scores have been extensively validated as measures of their respective constructs and consistently identify EF dysfunction in clinical populations with poor EF, such as children with attention-deficit/hyperactivity disorder (Gioia et al., 2000; Roth et al., 2014). BRIEF scores have also been used and validated in children who are DHH (e.g., Beer et al., 2014). BRIEF raw scores can be converted to *T*-scores using an age-based normative sample, such that higher scores indicate poorer EF. Two BRIEF subscales were chosen because they involve core subdomains of EF (e.g., Miyake et al., 2000) that have been identified as at-risk for delays in preschool-aged DHH children (Kronenberger et al., 2020): Inhibit (example item: “Does not think before doing”) and Working Memory (“When given three things to do, remembers only the first or last”). The LEAF is a behavior checklist that focuses on everyday child behaviors related to more cognitively-based EF behaviors in daily life (Castellanos et al., 2018). The LEAF demonstrated strong internal consistency, test-retest reliability, and validity as an EF measure, including significant correlations with scores on other EF behavior checklists and neurocognitive performance-based measures (Castellanos et al., 2018). Three LEAF subscales were selected because of evidence of delays in these EF domains in preschool-aged CI users (Kronenberger et al., 2014): Attention (example item: “Does not stay focused on learning material”), Working Memory (“Forgets things that he or she knew how to do a few hours or days before”), and Sustained Sequential Processing (“Loses track of step-by-step directions”). The LEAF yields raw scores, with higher scores corresponding to poorer EF. The LEAF and BRIEF scales capture overlapping yet complementary aspects of EF behavior because of their item choice and scale design (Castellanos et al., 2018). To create a comprehensive measure of children’s daily functioning and behaviors corresponding to EF, BRIEF and LEAF were combined into one composite score for analysis.

### Statistical analysis

Statistical analyses were performed using IBM SPSS v.28 (IBM Corporation, 2021); all *p*-values are two-tailed. For the language tests (PPVT, CASL, CELF), age norm-based (standard or scaled) scores were used in all analyses. To represent EF in daily life, an aggregate variable was created for each participant by averaging standardized z-scores [using the mean and standard deviation (SD) of the full sample) from *T*-scores of BRIEF Inhibit and Working Memory subscales and raw scores of LEAF Attention, Working Memory, and Sustained Sequential Processing subscales. Higher aggregate EF variable scores correspond to poorer EF. The T2 EF variable was missing for two DHH participants at T2 because parents failed to complete both LEAF and BRIEF. The selected EF scales all fall under the umbrella construct of EF, but are also theorized to work together and are connected cognitively to support functioning in daily life (Barkley, 2012). In addition, aggregation of the LEAF and BRIEF subscales into a single EF variable was supported by correlational and principal components analysis of T1 data. Concurrent full-sample bivariate Pearson correlations of included BRIEF (*T*-scores) and LEAF (raw scores) subscales ranged from *r* = 0.549 to 0.794 with a median correlation of *r* = 0.616 (full correlation tables are available upon request from the corresponding author). In a principal components analysis, a single component solution accounted for over half of the variance (Eigenvalue = 3.56), and all 5 T1 BRIEF and LEAF scores had loadings of 0.79 or greater on the component (median loading = 0.84). When all subsequent analyses were repeated with separate inhibitory control and working memory aggregate variables, the same trends reported below were found. Consequently, for parsimony, one EF aggregate variable was used in the remaining analyses.

Descriptive statistics (means, SDs, or frequency counts, as appropriate) were used to characterize the demographic and audiological characteristics of the TH and DHH samples, as well as the HA and CI subsamples within the DHH sample. Comparisons between samples and subsamples were carried out using independent samples *t*-tests for continuous data or chi-square tests for categorical data. To compare language and EF scores between the samples (TH vs. DHH) and subsamples (HA vs. CI) at both time points, analyses of covariance (ANCOVAs) were used, controlling for T1 child chronological age. Separately within each hearing group, predictive bivariate Pearson correlations were then performed between T1 language and T2 EF scores and T1 EF and T2 language scores to investigate longitudinal associations between EF and language separately for each hearing group.

Finally, hierarchical regression analyses (using the combined DHH and TH samples) were performed with each of the three T2 language scores as the criterion variable (3 separate equations for PPVT, CASL, and CELF). The first block of predictor variables (all entered into the equation regardless of statistical significance) consisted of hearing group, parental education, and the T1 language score corresponding to the language measure used as the criterion variable. The second variable block consisted of the T1 EF score (entered into the equation regardless of significance), to investigate the predictive association of T1 EF on T2 language, over and above the first block. Finally, the third variable block consisted of the product (interaction) of hearing group × T1 EF to investigate whether the T1 EF-T2 language association was moderated by hearing group; this term was retained in the final equation only if statistically significant, in order to reduce multicollinearity and adverse effects on power.

Conversely, hierarchical regression equations were also calculated predicting T2 EF from T1 language scores. The first block of predictor variables (all entered into the equation regardless of statistical significance) consisted of hearing group, parental education, and the T1 EF score. The second variable block consisted of the T1 language scores (PPVT, CASL, and CELF, each entered separately into the equation and tested for significance), to investigate the predictive association of T1 language on T2 EF, over and above the first block. Finally, the third variable block consisted of the three products (interactions) of hearing group x T1 language (PPVT, CASL, and CELF) to investigate whether the T1 language-T2 EF association was moderated by hearing group; this term was retained in the final equation only if statistically significant, in order to reduce multicollinearity and adverse effects on power.

## Results

### Longitudinal language/executive functioning scores and associations

Table 2 displays means and SDs of T1 and T2 language and EF for both hearing groups. As expected, TH children showed significantly better standard/scaled language scores than DHH children for all language measures at both T1 and T2 (T1 PPVT: *F* = 54.31, *p* < 0.001; T2 PPVT: *F* = 37.39, *p* < 0.001; T1 CASL: *F* = 12.74, *p* < 0.001; T2 CASL: *F* = 16.03, *p* < 0.001; T1 CELF: *F* = 23.21, *p* < 0.001; T2 CELF: *F* = 19.21, *p* < 0.001). TH children also had significantly lower (i.e., better) EF scores than DHH children at T1 (*F* = 11.11, *p* = 0.001), but not T2 (*F* = 3.02, *p* = 0.09). Children who use HAs also had significantly better language scores than children who use CIs at both timepoints (T1 PPVT: *F* = 5.53, *p* = 0.02; T2 PPVT: *F* = 4.73, *p* = 0.03; T2 CASL: *F* = 7.68, *p* = 0.008; T2 CELF: *F* = 8.92, *p* = 0.004), except for T1 CASL (*F* = 0.54, *p* = 0.47) and T1 CELF (*F* = 2.48, *p* = 0.12). Children who use HAs had significantly better T1 EF than children who use CIs (*F* = 7.00, *p* = 0.01), but did not show a significant difference in T2 EF (*F* = 2.35, *p* = 0.13).

**TABLE 2:** T1 and T2 language and EF descriptive statistics.

	TH	DHH (HA and CI)	HA	CI
Characteristics	M (SD)	M (SD)	M (SD)	M (SD)
T1 PPVT	116.88 (10.19)	97.30*** (17.62)	103.33 (16.57)	92.31* (17.15)
T2 PPVT	117.25 (12.83)	98.70*** (18.38)	104.58 (15.45)	93.83* (19.24)
T1 CASL	111.19 (12.55)	103.21*** (16.28)	105.00 (15.45)	101.72 (17.07)
T2 CASL	115.49 (10.54)	105.55*** (15.63)	111.75 (13.61)	100.41** (15.52)
T1 CELF	10.81 (2.84)	8.30*** (3.47)	9.08 (3.28)	7.66 (3.55)
T2 CELF	11.32 (3.02)	8.75*** (3.62)	10.25 (3.40)	7.52** (3.37)
T1 EF	–0.29 (0.78)	0.33** (1.12)	–0.10 (1.07)	0.68* (1.05)
T2 EF	–0.16 (0.97)	0.19 (1.02)	–0.06 (1.04)	0.39 (0.97)

Analyses of Covariance controlling for T1 child chronological age were used to compare between hearing groups; T1, timepoint 1; T2, timepoint 2, 10–14 months after T1; EF, executive functioning score; TH, typical hearing; DHH, deaf or hard-of-hearing; HA, hearing aid; CI, cochlear implant; PPVT, Peabody Picture Vocabulary Test–Fourth Edition, standard scores; CASL, Comprehensive Assessment of Spoken Language, Second Edition Sentence Comprehension subtest, standard scores; CELF, Clinical Evaluation of Language Fundamentals–Fifth Edition/Clinical Evaluation of Language Fundamentals Preschool–Second Edition, scaled scores. *p < 0.05, **p < 0.01, ***p < 0.001.

Table 3 reports predictive correlations between T1 language-T2 EF and T1 EF-T2 language. In the TH sample, no significant correlations were found between T1 language and T2 EF or T1 EF and T2 language. In contrast, DHH children showed significant correlations between T1 EF and two T2 language measures, T2 CASL (*r* = –0.353, *p* = 0.009) and T2 CELF (*r* = –0.381, *p* = 0.005; poorer EF associated with lower language scores), while no significant correlations were found between T1 EF and T2 PPVT or any T1 language measure and T2 EF.

**TABLE 3:** T1 and T2 longitudinal language/EF correlations.

	T1 EF			T2 EF	
	TH	DHH		TH	DHH
T2 PPVT	0.157	–0.224	T1 PPVT	–0.066	–0.089
T2 CASL	–0.028	–0.353**	T1 CASL	0.071	–0.169
T2 CELF	–0.143	–0.381**	T1 CELF	–0.163	–0.227

T1, timepoint 1; T2, timepoint 2 10–14 months after T1; EF, executive functioning score; TH, typical hearing; DHH, deaf or hard-of-hearing; PPVT, Peabody Picture Vocabulary Test–Fourth Edition, standard scores; CASL, Comprehensive Assessment of Spoken Language, Second Edition Sentence Comprehension subtest, standard scores; CELF, Clinical Evaluation of Language Fundamentals–Fifth Edition/Clinical Evaluation of Language Fundamentals Preschool–Second Edition, scaled scores. **p < 0.01.

### Longitudinal/predictive regressions

**FIGURE 1:**
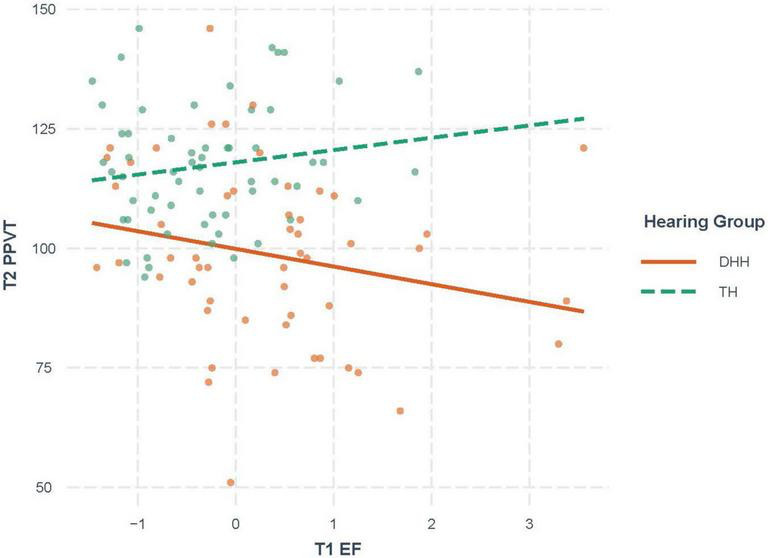
Interaction between TH and DHH children for the association of T1 EF and T2 PPVT. Children who are DHH demonstrate a marginally significant negative longitudinal association while the TH children do not show a significant association. T1, timepoint 1; T2, timepoint 2 10–14 months after T1; PPVT**,** Peabody Picture Vocabulary Test–Fourth Edition (standard scores); EF, executive functioning score; TH, typical hearing; DHH, deaf or hard-of-hearing.

Six hierarchical multiple linear regression analyses were conducted with both TH and DHH children combined in each analysis. For the first 3 analyses, the three T2 language variables served as criterion variables, and the primary predictor of interest was T1 EF, in order to investigate whether T1 EF predicted T2 language with T1 language, hearing group, and parental education controlled. In equations predicting the three language variables at T2 (Table 4), T1 language emerged as a significant predictor, and T1 EF added significantly to T1 language in predicting T2 language for CASL (*t* = –2.22, *p* = 0.03) and CELF (*t* = –2.67, *p* = 0.009). However, none of the hearing group × EF interaction terms were significant for the latter two outcomes. For PPVT, however, a significant hearing group x EF interaction was found (*t* = –2.71, *p* = 0.008). *Post hoc* analysis of the interaction using the Johnson-Neyman technique, as shown in Figure 1, revealed no significant relation between T1 EF and T2 PPVT for TH children (*t* = 0.99, *p* = 0.33), but a marginally significant negative relation for DHH children (*t* = –1.91, *p* = 0.06), such that lower (better) T1 EF scores were significantly related with higher (better) T2 PPVT scores.

**TABLE 4:** Hierarchical linear regressions predicting T2 language outcomes.

	T2 Language (Criterion)		
	PPVT	CASL	CELF
*Model 1*	0.73***	0.44***	0.49***
Hearing group	–0.04	–0.19*	–0.13
T1 language	0.81***	0.54***	0.65***
Parental education^a^	0.07	0.11	–0.04
*Model 2*	0.73***	0.46***	0.52***
Hearing group	–0.05	–0.14	–0.08
T1 language	0.81***	0.52***	0.62***
Parental education	0.07	0.12	–0.04
T1 executive functioning	0.04	–0.17*	–0.19**
*Model 3*	0.75***	NS	NS
Hearing group	–0.06		
T1 language	0.81***		
Parental education	0.06		
T1 executive functioning	0.23*		
Hearing group × T1 executive functioning	–0.23**		

Values for Model row are R^2^ (statistical significance is reported for the R^2^ value); values for variable rows are standardized regression weights. T1, timepoint 1; T2, timepoint 2 10–14 months after T1; PPVT, Peabody Picture Vocabulary Test–Fourth Edition (standard scores); EF, executive functioning score. ^a^Parental education was coded based on highest level of formal education: 1 = elementary school through 10 = doctorate degree. T1 Language = Language predictor variable (PPVT, CASL, or CELF) at T1 corresponding to T2 language criterion variable (e.g., PPVT at T1 for equation with PPVT at T2 as criterion variable). NS = Hearing Group × Executive Functioning terms were non-significant for equations predicting CASL and CELF. *p < 0.05, **p < 0.01, ***p < 0.001.

For the final 3 analyses, T2 EF served as the criterion variable, and separate analyses were conducted with each T1 language variable entered in the second block to test prediction of T2 EF from T1 language with T1 EF, hearing group, and parental education controlled. Table 5 reports the regression analyses with T2 EF as the dependent variable and separate tests for each language score in Model 2 (three models). For all three models, the only significant main effect was T1 EF. No language score significantly predicted T2 EF in Model 2, and the addition of the hearing group x T1 language interaction in Model 3 did not significantly improve model fit.

**TABLE 5:** Hierarchical linear regressions predicting T2 executive functioning.

	T2 Executive functioning (Criterion)
*Model 1*	0.63***
Hearing group	–0.06
T1 executive functioning	0.81***
Parental education^a^	0.07
*Model 2 (T1 PPVT as Predictor)*	0.63***
Hearing group	–0.04
T1 executive functioning	0.82***
Parental education	0.06
T1 PPVT	0.05
*Model 2 (T1 CASL as Predictor)*	0.63***
Hearing group	–0.06
T1 Executive functioning	0.82***
Parental education	0.06
T1 CASL	0.03
*Model 2 (T1 CELF as Predictor)*	0.64***
Hearing group	–0.09
T1 Executive functioning	0.80***
Parental education	0.08
T1 CELF	–0.10

Model 1 is the same for each language variable tested in Model 2. Each Model 2 shown is for one of the language variables (PPVT, CASL, CELF) predicting T2 Executive Functioning. Values for Model rows are *R*^2^; values for variable rows are standardized regression weights. Model 3 is not shown because all Hearing Group × Language product (interaction) variables were non-significant (*p* > 0.10) and did not meet criteria for model entry. T1, timepoint 1; T2, timepoint 2 10–14 months after T1; PPVT, Peabody Picture Vocabulary Test–Fourth Edition (standard scores); EF, executive functioning score. ^a^Parental education was coded based on highest level of formal education: 1 = elementary school through 10 = doctorate degree. ***p < 0.001.

## Discussion

The purpose of this study was to examine the predictive, reciprocal associations between EF and spoken language over a 1-year period in DHH and TH samples of preschool-aged children at entrance into the study. Consistent with our first hypothesis, DHH children demonstrated longitudinal associations between EF and measures of later language in correlational analyses as well as regression analyses even after controlling for baseline language, whereas evidence for the reverse was not found. Consistent with our second hypothesis, correlations for T2 higher-order language (comprehension and following directions) and T1 EF were statistically significant in the DHH sample but not in the TH sample, and for T2 receptive vocabulary, the significant interaction term for hearing group and T1 EF demonstrated a stronger association between T1 EF and T2 vocabulary in the DHH group than in the TH group. These results are the first to demonstrate that everyday parent-rated EF behaviors predict basic (vocabulary) and higher-order (comprehension, following directions) language development 1 year later in preschool-aged DHH children even after accounting for baseline language skills. The current study was also the first longitudinal study to focus exclusively on parent-rated EF behaviors in daily life; prior work has focused either exclusively (Jones et al., 2020) or partly (Kronenberger et al., 2020) on individual ability testing of EF in the office/lab setting, which shares method variance with individually administered language tests.

The finding in this study that T1 EF significantly predicted T2 language in preschool-aged DHH children, but not the reverse, is similar to results obtained by Kronenberger et al. (2020), providing further evidence of the importance of early EF for later language development of DHH children at young ages. On the other hand, this finding contrasts with that of Jones et al. (2020), who found that T1 language predicted T2 EF, but not the reverse, in their sample of DHH children. The discrepancy of these findings may be due to the different ages of the children in these studies. The current study (ages 3–8 years) and the study of Kronenberger et al. (2020; ages 3–6 years) included much younger (many preschool-aged) children than Jones et al. (2020) (6–12-year-old children). Language learning is more rapid earlier in development, increasing the potential for factors to influence its development at younger ages. In support of this hypothesis, Jones et al. (2020) report a path coefficient of 0.88 from their T1 vocabulary to T2 vocabulary score in their older sample, indicating extremely high language stability and leaving little unexplained variance for EF (or any other variable) to account for. On the other hand, in the current younger sample, the models with T1 CASL and CELF as predictors accounted for 44–49% of the variance in their respective T2 scores, leaving over half of the T2 language variance available for explanation by other contributing factors.

Another potential explanation for the discrepancy between the current study and Jones et al. (2020) may be the domains of language processing assessed. The current study assessed receptive language and included one measure of word knowledge (vocabulary) and two measures of higher-order language/discourse processing involving concept formation, integration of linguistic meaning, and memory (comprehension and following directions). In contrast, Jones et al. (2020) focused on expressive single word vocabulary as their only measure of language and did not include any higher-order language measures. Of note, a cross-sectional study reporting that language accounted for EF differences between hearing groups—but not the reverse (EF accounting for language)—also used only single word expressive vocabulary as the sole measure of language in a sample of school aged children 5–11 years of age (Botting et al., 2017).

In the current study, the correlation between T1 EF and the T2 measure of single-word vocabulary (PPVT) was not significant (Table 3), nor was the main effect of T1 EF predicting T2 PPVT in Model 2 of the hierarchical regression (Table 4), although the full regression equation for PPVT (including the interaction block) did indicate T1 EF as a significant predictor for T2 PPVT for the DHH sample. On the other hand, T2 higher-order language measures were significantly predicted by T1 EF not only in the current study but also in another prior longitudinal study of children with CIs, using the Preschool Language Scale-2 to assess higher-order language (Kronenberger et al., 2020). This pattern of findings suggests that more basic vocabulary knowledge scores may be more stable over time and less influenced by earlier EF than higher-order language, which was predicted by earlier EF in the current study and in other studies. Higher-order language processing is at greater risk for delay, more dependent on EF, and not fully explained by vocabulary skills in DHH samples, suggesting that EF may have a greater longitudinal role in development of higher order language than basic vocabulary skills (Kronenberger and Pisoni, 2019).

It is also possible that some domains of language may contribute more to EF development than others, allowing for a predictive association of language explaining later EF outcomes. Expressive vocabulary as measured in Botting et al. (2017) and Jones et al. (2020), for example, may better account for the contribution of language to EF development. One hypothesis for this mediating effect of language on EF development may be that expressive language is used to regulate and direct thinking and behavior in a goal-directed manner (Zelazo and Frye, 1998). On the other hand, receptive vocabulary, used in this study, is a measure of word understanding, not use, and so may better reflect the ability of EF skills to facilitate hearing, learning, and understanding surrounding language during processing. Alternatively, single-word vocabulary (whether expressive or receptive) may be a better predictor of later EF skills than higher-order language skills. In addition to Jones et al. (2020) finding that single word expressive vocabulary predicting later EF skills, Kronenberger et al. (2020) found that single word receptive vocabulary (PPVT scores) predicted one measure of verbal short-term/working memory (digit span forward) in preschoolers, whereas a higher-order language measure did not. Overall, this pattern of findings across different studies suggests that developmental stage, domain of language, and domain of EF should be considered when examining the predictive longitudinal associations between language and EF; simple, broad, unidirectional effects do not appear to accurately represent the complexity of reciprocal contributions of language and EF skills (Kronenberger and Pisoni, 2020).

An additional consideration in integrating results across studies is the measurement modality used for language and EF. Most of the early investigations of EF skills in DHH children with CIs or HAs relied on individually administered tests of ability in a controlled (lab, office, clinic) setting to operationalize EF (Figueras et al., 2008; Pisoni et al., 2010), while some later research has assessed EF using parent-report behavior checklists (Kronenberger et al., 2014). A large body of research has demonstrated that these different measurement modalities produce only modestly (albeit significantly) correlated EF scores (Barkley, 2012; Toplak et al., 2013), making the measurement modality a crucial consideration in application and interpretation of EF results. Because almost all language tests are individually administered behavioral performance tests in a controlled setting, language tests share method variance with individually administered, office/lab-based EF tests, and some of their shared variance may therefore reflect the effects of shared administrative methodology (e.g., good ability test-takers vs. poor ability test-takers; focus/motivation during individually administered tests of any ability, including language or EF). Parent-report questionnaire measures of EF do not share this method variance with individually administered, office/lab-based language tests, providing an advantage to studies such as the current one, which use EF questionnaires. On the other hand, parent-report questionnaires suffer from their own limitations, including parental response bias, variation in parent awareness/familiarity with child behavior, and parent personality factors. Hence, because any measurement methodology has limitations, integration of findings using different measurement modalities offers the greatest potential for understanding associations between constructs (Holmbeck et al., 2002). As a result, this study reports important novel information about EF and language development in DHH children by focusing on a relatively underused method for assessing EF skills—parent-report questionnaires.

Our second hypothesis, that the EF-language association would be stronger in DHH than in TH children, was partially supported by study findings. We expected a stronger EF-language association in DHH children than in TH children because language processing in TH children is typically fast and automatic, requiring less scaffolding by EF skills (Posner and Snyder, 2004). In contrast, DHH children may use more cognitive effort and working memory resources (components of EF) in the context of slow-effortful language processing to compensate for underspecified, coarse-coded phonological-lexical representations of words in memory (Rönnberg et al., 2013; Pichora-Fuller et al., 2016). Furthermore, when auditory access or linguistic representations are disrupted, as can happen for DHH children, the use of available EF in detection, processing, and encoding language may be more important for DHH than for TH children (Houston et al., 2020). Therefore, we would expect that the relation between EF and language to be stronger in children who are DHH than in TH children. Consistent with these predictions, results of an earlier experimental study demonstrated that DHH children with CIs are more reliant on a specific EF subdomain, verbal working memory, during language processing, than TH peers (Kronenberger et al., 2018).

In the current study, we found statistically significant correlations between T1 EF and T2 higher-order language (CASL and CELF) only in the DHH sample and not in the TH sample, consistent with our hypothesis of stronger EF-language associations in DHH children. However, *z*-tests comparing these correlations across the DHH and TH samples failed to reach statistical significance [*z* = 1.74 (*p* = 0.10) and 1.32 (*p* = 0.19) for CASL and CELF, respectively]. Furthermore, the hearing group x EF interaction predicting language outcome was significant only for the PPVT, such that EF was a stronger predictor of PPVT scores 1 year later in the DHH group than in the TH group. Thus, despite some indications of a stronger role for EF in language outcomes for DHH children, results were not consistently statistically significant. Future research with larger samples is recommended to further investigate this association, because results could have been affected by insufficient power.

Examining language and EF outcomes between groups revealed that TH children had significantly better T1/T2 language and T1 EF scores than DHH children when controlling for age differences between groups. This is consistent with extensive previous literature documenting language and EF delays, difficulties, and variability in DHH children who use auditory-oral spoken language as their mode of communication (e.g., Niparko et al., 2010; Kronenberger et al., 2014). Auditory and language development are inextricably related with neurocognitive development, especially early in life when neural development and organization are dependent on a wealth of sensory experiences (Kronenberger and Pisoni, 2018); any interruptions, delays, or distorted auditory or language input as a result of hearing loss would be expected to introduce more variability into related development in DHH children than TH children. One example for spoken language development is a prolific and ongoing research area documenting that the amount of parental language spoken in the home plays a significant role in later language development (e.g., Hart and Risley, 2003). Children who are DHH often inconsistently overhear language spoken in their environment that is not directed at them (McCreery et al., 2015). Overhearing contributes to language development and DHH children’s altered auditory experience with overhearing can differentially influence their development. In relation to EF variability in DHH children, the primary hypotheses for this difference lies in early auditory (e.g., Kral et al., 2016) and/or language deprivation (e.g., Hall, 2017) due to hearing loss that causes cascading neurocognitive effects during time-sensitive periods of neural development and organization (Kronenberger and Pisoni, 2018). In this study, the focus was on how DHH children who primarily use auditory-oral spoken language utilize their available EF skills in relation to later language learning, given underlying population variability.

It is also worth noting that TH children performed approximately one standard deviation above the mean on all language measures except the CELF. TH children as a group had significantly higher parental education and household income levels, although the differences between groups functionally represented a difference in type of college degree or about $15,000 per year in household income. Despite our attempts to use similar recruitment strategies for DHH and TH samples, use of a volunteer sampling strategy likely resulted in a higher-than-average functioning TH sample. In order to address parental education differences between samples, we controlled for parental education in our regression analyses; we did not also control for family income because of the strong association between parental education and family income in the study sample (*r* = 0.425, *p* < 0.001).

The DHH sample in this study was heterogeneous in several ways, most notably in device used. DHH participants used either HAs or CI(s), and varied in number of CIs (one or two) and audiological functioning (Table 1). The use of a DHH sample comprised of both HA and CI users has both advantages and limitations. One advantage is the investigation of outcomes across a wide range of audiological functioning and intervention history, particularly for children with HAs, who are an understudied clinical population (Donahue, 2007). Recent research efforts have begun to document more extensive data on language and EF development in children with HAs, showing cross-sectional associations of language with BRIEF WM and Inhibit (McCreery and Walker, 2022). An additional advantage of a combined HA/CI sample is the potential to compare outcomes. Studies examining language and EF in samples comprised of children who use HAs and children who use CIs are relatively rare. In this sample, children who use HAs tended to show better language and EF outcomes than children who use CIs, consistent with differing degrees of hearing loss and intervention. However, children who use HAs also tended to demonstrate lower scores and more variability than children with TH, extending previous findings as to the research and clinical needs of these children (Stiles et al., 2012). The primary limitation of a combined sample of HA and CI users is the added heterogeneity in outcomes and possibility of different associations with outcomes in HA vs. CI users. In order to have sufficient power for predictive/longitudinal analyses in the current study, HA and CI users were combined into a single DHH sample, as has been done in previous studies (e.g., Figueras et al., 2008). However, future research with larger sample sizes allowing for comparison of HA and CI users is recommended.

### Limitations

The results of this study should be interpreted in light of some methodological limitations, in addition to the use of a combined sample of HA/CI users discussed earlier. The TH and DHH samples differed along several demographic dimensions (age, parent education, parent income), although these dimensions were statistically controlled in analyses. Additionally, while longitudinal/predictive models constrain causal directions somewhat (e.g., a T2 variable cannot retrospectively cause a T1 variable), causality cannot be definitively concluded from predictive correlations or regressions alone in the absence of experimental manipulation. Thus, it is possible that third variables or mediating variables could affect the predictive associations found between EF and language in this study. Furthermore, while the sample size of 53 DHH and 59 TH participants is large in the context of previous studies of preschool-aged DHH children, it may not have provided sufficient power to detect small to medium effect sizes. Particularly for TH children, larger sample sizes may have produced greater variability and greater power to detect language-EF associations, and therefore non-significant results for TH children should be interpreted with caution. Smaller sample sizes may be sufficient to detect significant effect sizes in DHH children because of the larger associations between EF and language. Finally, while not a limitation *per se*, the results of this study should be interpreted in the context of the EF measurement modality of parent-report questionnaires and the specific use of two questionnaires—the BRIEF and LEAF. We selected these questionnaires and subscales because of prior results demonstrating their validity and importance in characterizing EF in samples of DHH children (Pisoni et al., 2010). Questionnaires with other content or other EF domains may produce different results.

## Conclusion and future directions

Findings in this study documented the first longitudinal, predictive relations of parent-rated EF behaviors in daily life with later language abilities when accounting for earlier language over a period of 1 year in a sample including preschool-aged DHH children. These results support the potential malleability of language development in young DHH children depending on earlier EF at preschool ages. In addition to enhancing our understanding of EF effects on language development in DHH children, these findings have significant clinical implications by suggesting that interventions to improve EF in everyday behavior at early ages may provide an opportunity to enhance language outcomes in DHH children. Previous research and clinical work have suggested early and continued EF intervention in DHH children can scaffold later EF and language development (Robbins and Kronenberger, 2021); these results support that expectation and should be further investigated. Future work should also continue to explore the mechanistic process by which EF supports language in young DHH children and should test the impact of improving EF on language outcomes in the DHH population.

## Data availability statement

The raw data supporting the conclusions of this article will be made available by the authors, without undue reservation.

## Ethics statement

The studies involving human participants were reviewed and approved by the Biomedical Sciences Institutional Review Board at the Ohio State University. Written informed consent to participate in this study was provided by the participants’ legal guardian/next of kin.

## Author contributions

IJ, WK, DP, and RH contributed to conception and design of the study. RH organized the database. IJ and WK performed the statistical analysis. IJ wrote the first draft of the manuscript. RH, WK, and DP contributed edits to the manuscript. All authors contributed to manuscript revision, reading, and approving the submitted version.
